# Overexpression of NOX4 predicts poor prognosis and promotes tumor progression in human colorectal cancer

**DOI:** 10.18632/oncotarget.16829

**Published:** 2017-04-04

**Authors:** Xiao-Lu Lin, Li Yang, Seng-Wang Fu, Wen-Feng Lin, Yun-Jie Gao, Hao-Yan Chen, Zhi-Zheng Ge

**Affiliations:** ^1^ Division of Gastroenterology and Hepatology, Key Laboratory of Gastroenterology and Hepatology, Ministry of Health, Renji Hospital, School of Medicine, Shanghai Jiao Tong University, Shanghai Institute of Digestive Disease, Shanghai 200001, China; ^2^ Division of Gastroenterology and Hepatology, Key Laboratory of Gastroenterology and Hepatology, Ministry of Health, State Key Laboratory for Oncogenes and Related Genes, Renji Hospital, School of Medicine, Shanghai Jiao Tong University, Shanghai Institute of Digestive Disease, Shanghai 200001, China; ^3^ Department of Gastroenterology, Shanghai General Hospital, Shanghai Jiao Tong University School of Medicine, Shanghai 200001, China

**Keywords:** colorectal carcinoma, NOX4, prognosis, proliferation, metastasis

## Abstract

NADPH oxidase 4 (NOX4), a major source of reactive oxygen species (ROS) production, has been increasingly reported to be involved in tumorigenesis and/or tumor progression, but limited data are available regarding the role of NOX4 in colorectal carcinoma (CRC). We retrieved six independent investigations from Oncomine database and found that NOX4 is highly expressed in CRC tissues compared with corresponding normal controls. Similar results were also found in clinical specimens at both mRNA and protein levels. Immunohistochemical analysis indicated that NOX4 overexpression was highly correlated with T classification, N classification, distant metastasis, and poor prognosis of CRC patients, which was also confirmed by GSE14333 and GSE17536 datasets from the Gene Expression Omnibus. Furthermore, we demonstrated that when NOX4 expression was knocked down by siRNAs, cell proliferation, cell-cycle and apoptosis, migration and invasion were significantly altered in CRC cell lines HCT116 and LOVO. Meanwhile, NOX4 promoted cancer cell proliferation and apoptosis, migration and invasion by regulating the expression of relevant genes. By these approaches we aim to elucidate NOX4 may be a reliable prognostic factor or therapeutic target in CRC.

## INTRODUCTION

Colorectal cancer (CRC) is the third most common type of malignancy in men and the second in women worldwide, with over 1.2 million new cases recorded and about 600,000 deaths per year [1–3]. The prognoses of CRC patients between early and advanced stages can vary from a 5-year survival rate of 93% at Stage I to 8% at Stage IV [4]. A majority of advanced cases ultimately die after surgery as a result of cancer recurrence or metastasis. Despite a mass of efforts have been made to improve the diagnosis and therapy of CRC, the clinical outcome of CRC is still unsatisfactory [5]. Consequently, more studies are needed to explore the potential mechanism and detect effective diagnostic and prognostic biomarkers to prolong the lives of CRC patients.

Reactive oxygen species (ROS) have recently been recognized as important intracellular signals in various physiological and pathological processes. High level of ROS can induce cell senescence, apoptosis and death due to their cytotoxic and mutagenic potential. In contrast, ROS at low level serve as regulators of cell growth, differentiation, apoptosis and gene expression [6]. Tumors always exhibit excessive and persistent elevation of ROS, and several studies have demonstrated that ROS may be associated with human carcinogenesis [7, 8].

As a major source of ROS generation, the NADPH oxidase (NOX) family, including seven members (NOX1–5 and DUOX1–2), is attracting increasing attention for the correlation with cancer development and progression [9]. NOX1 is essential for oncogenic Ras transformation phenotype [10], and NOX5 is involved in cell viability of prostate cancer cells [11]. Among the members of NOX family, NOX4 is the most frequently expressed NOX isoform in terms of its relevance with cancer. Overexpression of NOX4 has been found in diverse types of solid tumors, such as prostate cancer [12], glioblastoma [13], liver cancer [14], and melanoma [15]. These studies also demonstrated the involvement of NOX4 in cancer proliferation, metastasis, apoptosis, tumorigenic transformation, and radiation resistance. However, the role of NOX4 in colorectal carcinoma and the underlying molecular mechanism responsible for its involvement in tumorigenesis and/or tumor progression are far from clear. The clinicopathological and prognostic significance of NOX4 expression patterns in CRC patients is still unknown.

In the present study, we profiled the expression status of NOX4 at both the mRNA and protein levels. We further analyzed the relationship between NOX4 expression and clinicopathologic parameters including prognostic significance, and explored the biological function and molecular mechanism of NOX4 in CRC patients in the light of GeneSet Enrichment Analysis (GSEA) on The Cancer Genome Atlas (TCGA) dataset.

## RESULTS

### Expression of NOX4 is significantly up-regulated in CRC

**Figure 1:**
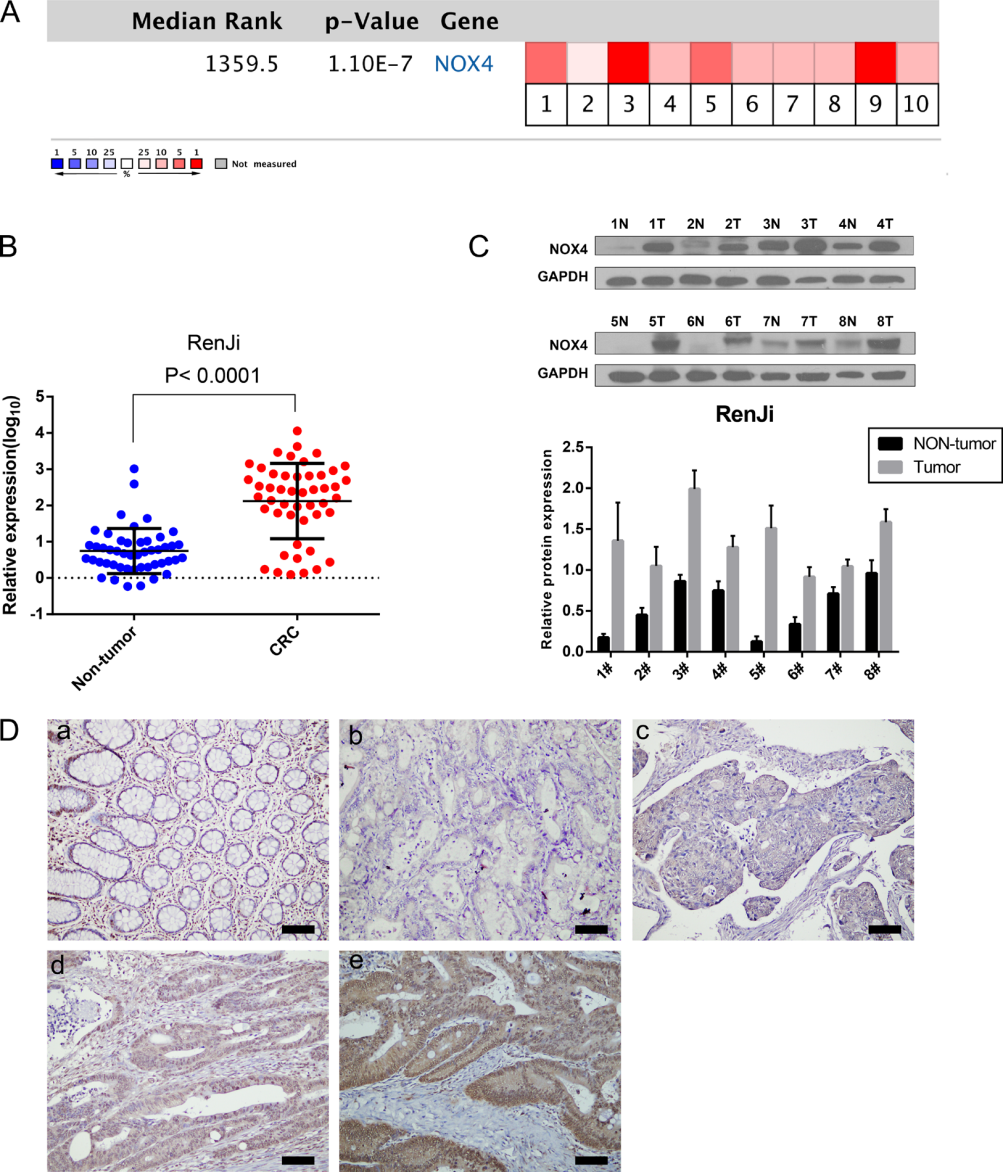
NOX4 expression is up-regulated in CRC (**A**) A meta-analysis of NOX4 gene expression from six Oncomine databases where colored squares indicate the median rank for NOX4 (vs. Normal tissue) across 10 analyses. Gaedcke Colorectal (1), Hong Colorectal (2), Kaiser Colon (3, 4), Skrzypczak Colorectal (5), Skrzypczak2 Colorectal (6), and TCGA (7–10). The *P* value is given for the median-rank analysis. (**B**) NOX4 mRNA expression was examined by RT-PCR and normalized to 18S expression (*P* < 0.0001, paired *t*-test). (**C**) The image of western blotting analyses of NOX4 protein expression in eight pairs of CRC samples, GAPDH was used as a loading control. (**D**) Immunohistochemical representative images of NOX4 expression in CRC samples compared with corresponding normal tissue. (a) Normal tissue; (b) CRC, scored as (−); (c) CRC, scored as (+); (d) CRC, scored as (++); (e) CRC, scored as (+++) (scale bar, 100 μm).

To assess the role of NOX4 in CRC, we first analyzed six independent microarray datasets from Oncomine database [16–19], and revealed statistically significant overexpression of NOX4 in the majority of CRC tissues compared with adjacent non-neoplastic controls (Table 1). The median rank of NOX4 in up-regulated genes of CRC was 1359.5 based on a meta-analysis across the six datasets, including 10 analyses using the Oncomine algorithms [20] (699 samples, *P* = 1.10E-7, Figure 1A). We further validated the expression level of NOX4 in 56 pairs of CRC specimens (tumor and corresponding nontumor tissues) by RT-PCR and western blot as shown in Figure 1B and 1C. As expected, the mRNA and protein expression of NOX4 was markedly up-regulated in CRC tissues compared with their nontumor counterparts.

**Table 1:** Detailed information about the 6 public expression datasets of Oncomine database about NOX4 in CRC

Datasets (sample size)	Comparison groups	Fold Change	*P* value	Overexpression Gene Rank
TCGA colorectal (237)	Colon Mucinous Adenocarcinoma vs. Normal	9.646	1.17E-14	107 (in top 1%)
	Cecum Adenocarcinoma vs. Normal	4.537	1.49E-08	1547 (in top 8%)
	Colon Adenocarcinoma vs. Normal	6.371	9.07E-13	1675 (in top 9%)
	Rectal Adenocarcinoma vs. Normal	5.171	9.50E-12	1815 (in top 9%)
Kaiser Colon (105)	Colon Adenocarcinoma vs. Normal	2.063	1.46E-12	154 (in top 1%)
	Colon Mucinous Adenocarcinoma vs. Normal	3.678	4.27E-05	1640 (in top 9%)
Gaedcke Colorectal (130)	Rectal Adenocarcinoma vs. Normal	3.145	3.04E-27	357 (in top 2%)
Skrzypczak Colorectal (105)	Colorectal Carcinoma vs. Normal	2.495	1.26E-08	732 (in top 4%)
Skrzypczak Colorectal2 (40)	Colon Carcinoma vs. Normal	7.523	2.05E-07	1172 (in top 6%)
Hong Colorectal (82)	Colorectal Carcinoma vs. Normal	2.416	4.37E-04	4692 (in top 24%)

Moreover, we measured NOX4 expression in a CRC tissue microarray (*n* = 82, paraffin-embedded tissues). Positive staining of NOX4 was mainly distributed in the cytoplasm and nucleus of CRC cells. Among 82 cases of CRC, 46 (56.1%) CRC tissues showed high NOX4 expression (NOX4 ++ or NOX4 +++), whereas the remaining 36 cases (43.9%) had a low level of NOX4 expression (NOX4− or NOX4+). Representative IHC staining patterns for NOX4 in CRC are shown in Figure 1D. Statistical analysis also revealed that CRC tissues expressed a significantly higher level of NOX4 protein than adjacent non-malignant tissues (Table 2).

**Table 2:** NOX4 expression in colorectal cancerous and paracancerous tissues

		Tissue	
immunohistochemical grade	CRC tissue (*n* = 82, 100%)	paracancerous tissues (*n* = 82, 100%)	*P* value
negative (−)	4 (4.8)	35 (42.7)	< 0.001
weakly positive (+)	32 (39.0)	39 (47.6)	
median strongly positive (++)	34 (41.5)	6 (7.3)	
strongly positive (+++)	12 (14.6)	2 (2.4)	

### Association of NOX4 expression with clinicopathological features and prognosis in CRC patients

Next, the association of NOX4 high-expression with clinicopathological features in 82 CRC samples with informative IHC was statistically analyzed. As summarized in Table 3, increased expression of NOX4 significantly associated with T classification (*P* = 0.005), lymph node metastasis (*P* = 0.026), distant metastasis (*P* = 0.014). Interestingly, we also observed that T stage significantly associated with NOX4 expression in TCGA database (*P* = 0.011, Figure 3A). Other clinical parameters, such as age, gender, tumor size, tumor location and histological differentiation, were barely associated with high NOX4 protein expression.

**Table 3:** Association of NOX4 expression with the clinicopathological characteristics of CRC

			NOX4 expression		
clinicopathological feature	Category	Total (%)	Low expression (*n* = 36, 43.9%)	High expression (*n* = 46, 56.1%)	*P* value
**Sex**	Male	47 (57.3)	22 (46.8)	25 (53.2)	
	Female	35 (42.7)	14 (40.0)	21 (60.0)	0.539
**Age(years)**	< 65	35 (42.7)	17 (48.6)	18 (51.4)	
	≥ 65	47 (57.3)	19 (40.4)	28 (59.6)	0.462
**Tumor size**	< 5 cm	35 (42.7)	17 (48.6)	18 (51.4)	
	≥ 5 cm	47 (57.3)	19 (40.4)	28 (59.6)	0.462
**Tumor location**	Right hemicolon	44 (53.7)	17 (38.6)	27 (61.4)	
	Left hemicolon	38 (46.3)	19 (50.0)	19 (50.0)	0.301
**T classification**	T1	1 (1.2)	0 (0.0)	1 (100.0)	
	T2	4 (4.9)	4 (100.0)	0 (0.0)	
	T3	55 (67.1)	28 (50.9)	27 (49.1)	
	T4	22 (26.8)	4 (18.2)	18(81.8)	**0.005**
**Lymph node metastasis**	Absent	41 (50.0)	23 (56.1)	18 (43.9)	
	Present	41 (50.0)	13 (31.7)	28 (68.3)	**0.026**
**Distant metastasis**	Absent	73 (89.0)	36 (49.3)	37 (50.7)	
	Present	9 (11.0)	0 (0.0)	9 (100.0)	**0.014**
**Histological differentiation**	Well/moderate	60 (73.2)	25 (41.7)	35 (58.3)	
	poor	22 (26.8)	11 (50.0)	11 (50.0)	0.500

Values in parentheses indicate percentage values. Bold values indicate statistical significance, *P* < 0.05.

**Figure 2:**
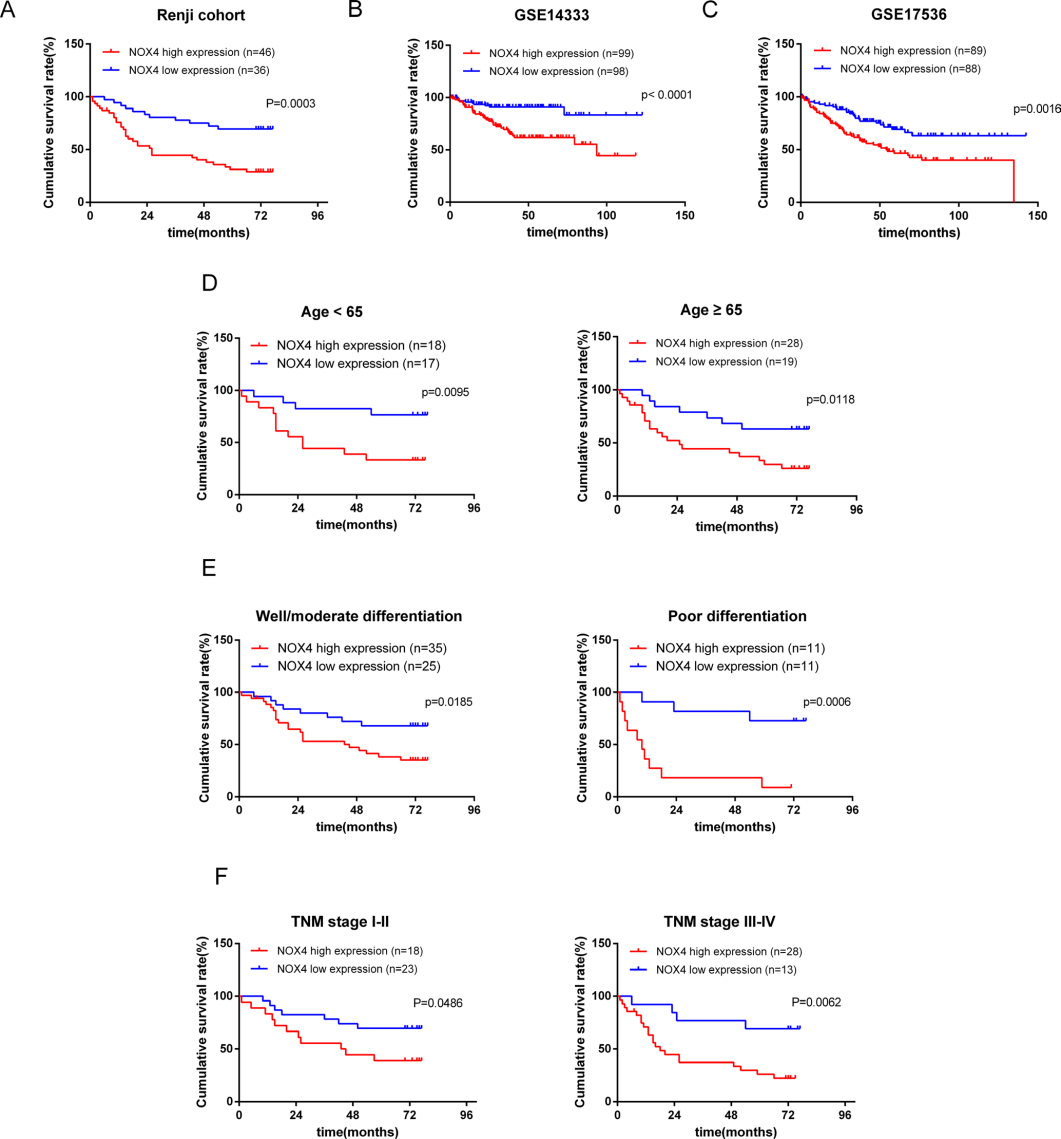
Kaplan-Meier analysis of overall survival in CRC patients (**A**) Overall survival analysis of 82 CRC patients with different NOX4 protein expression in Renji cohort. (Log-rank test: *P* = 0.0003). (**B**, **C**) The association of NOX4 expression with patient survival was conducted in GSE14333 and GSE17536 dataset from GEO, respectively. (**D–F**) Comparisons of overall survival between NOX4 high expression and NOX4 low expression in CRC patients regardless of age, histological differentiation and TNM stage.

To further investigate the prognostic significance of NOX4 expression in patients with CRC, we used Kaplan-Meier analysis to evaluate the correlation between NOX4 expression and the survival curve. As shown in Figure 2, patients with high NOX4 expression showed a significantly shorter cancer-specific survival than those with a low level of NOX4 expression (log-rank test, *P* = 0.0003, Figure 2A), clearly demonstrating that high NOX4 expression was associated with a shorter survival rate. In addition, we analyzed the correlation of NOX4 expression with overall survival in GSE14333 and GSE17536 with a total of 197, 177 cases enrolled from the GEO datasets. The results showed that NOX4 expression was highly associated with CRC patients’ overall survival (log-rank test, *p* < 0.001, *p* = 0.0016, Figure 2B and 2C). Besides, we further analyzed the overall survival time in different sub-groups according to age, histological differentiation and TNM stage, and it was demonstrated that overall survival was shorter in CRC patients with higher NOX4 expression independent of age, histological differentiation and TNM stage (Figure 2D and 2F).

**Figure 3:**
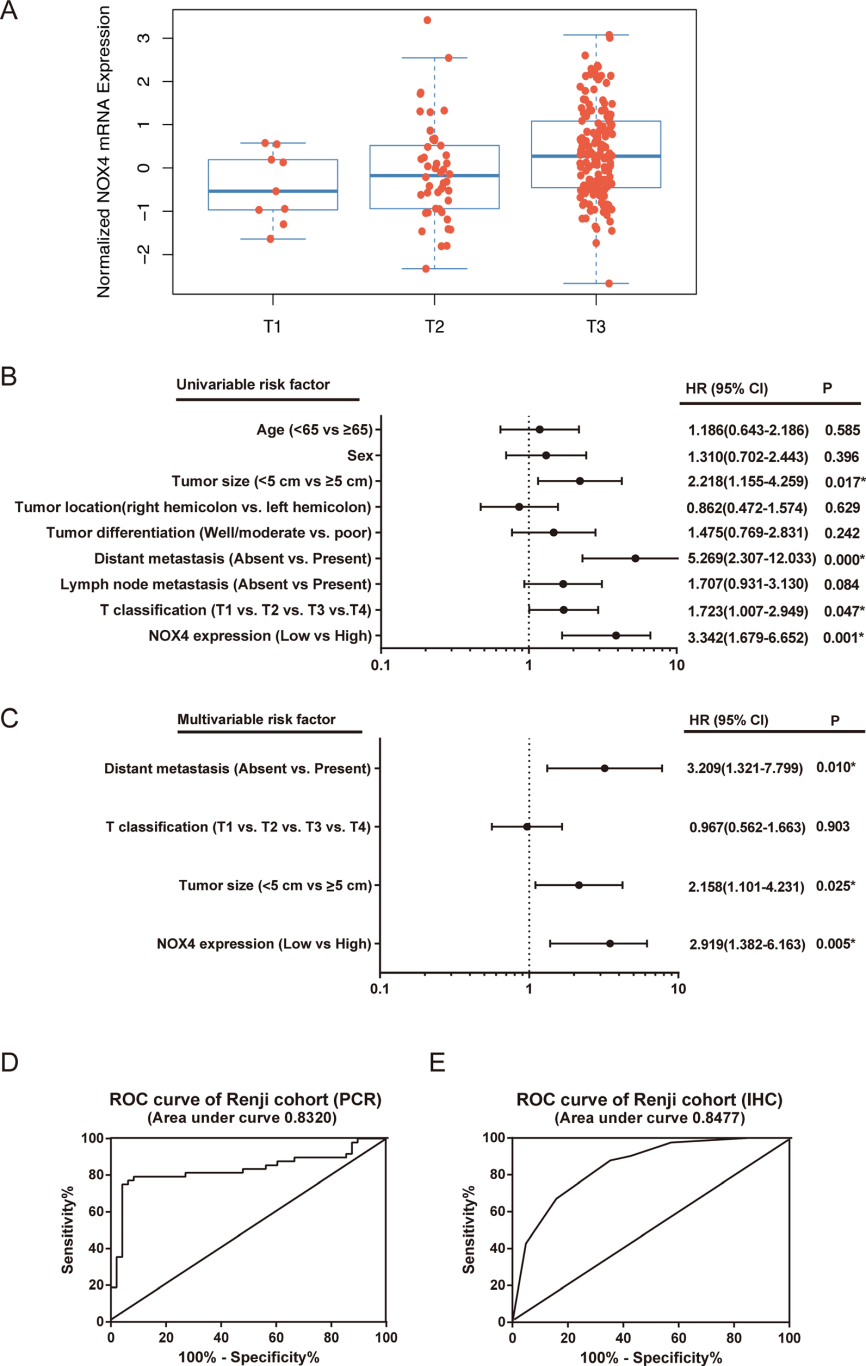
The potential value of NOX4 expression in predicting CRC patient prognosis (**A**) T stage significantly associates with NOX4 expression in TCGA database. The number of T1, T2, and T3 is 9, 46 and 150, respectively (*P* = 0.011). (**B**, **C**) The forest plot showed the correlation between CRC patient overall survival and NOX4 expression as well as other clinical characteristics by using univariable (B) and multivariable (C) analyses. (**D**, **E**) The Potential predictive value of NOX4 expression according to ROC curve analysis from two retrospective cohorts. (A) RT-PCR; (B) IHC.

Univariate and multivariate Cox regression analyses were performed to confirm the possibility that NOX4 could be useful as an independent risk factor for poor prognosis in the 82 cases of CRC. Univariate analysis showed that NOX4 expression, tumor size, T classification and distant metastasis were significantly associated with a poorer clinical outcome in CRC cases (Table 4, Figure 3B). Meanwhile, multivariate analysis identified that NOX4 expression, tumor size and distant metastasis were independent prognostic factors in patients with CRC (Table 3, Figure 3C). Furthermore, conventional receiver operating characteristic (ROC) analysis was used to investigate the prediction value of NOX4 expression. Results demonstrated that NOX4 was a practical predictor in CRC, with an area under curve (AUC) of 0.8320 and 0.8477 in 48 (RT-PCR) and 82 (IHC) paired patients, respectively (Figure 3D and 3E).

**Figure 4:**
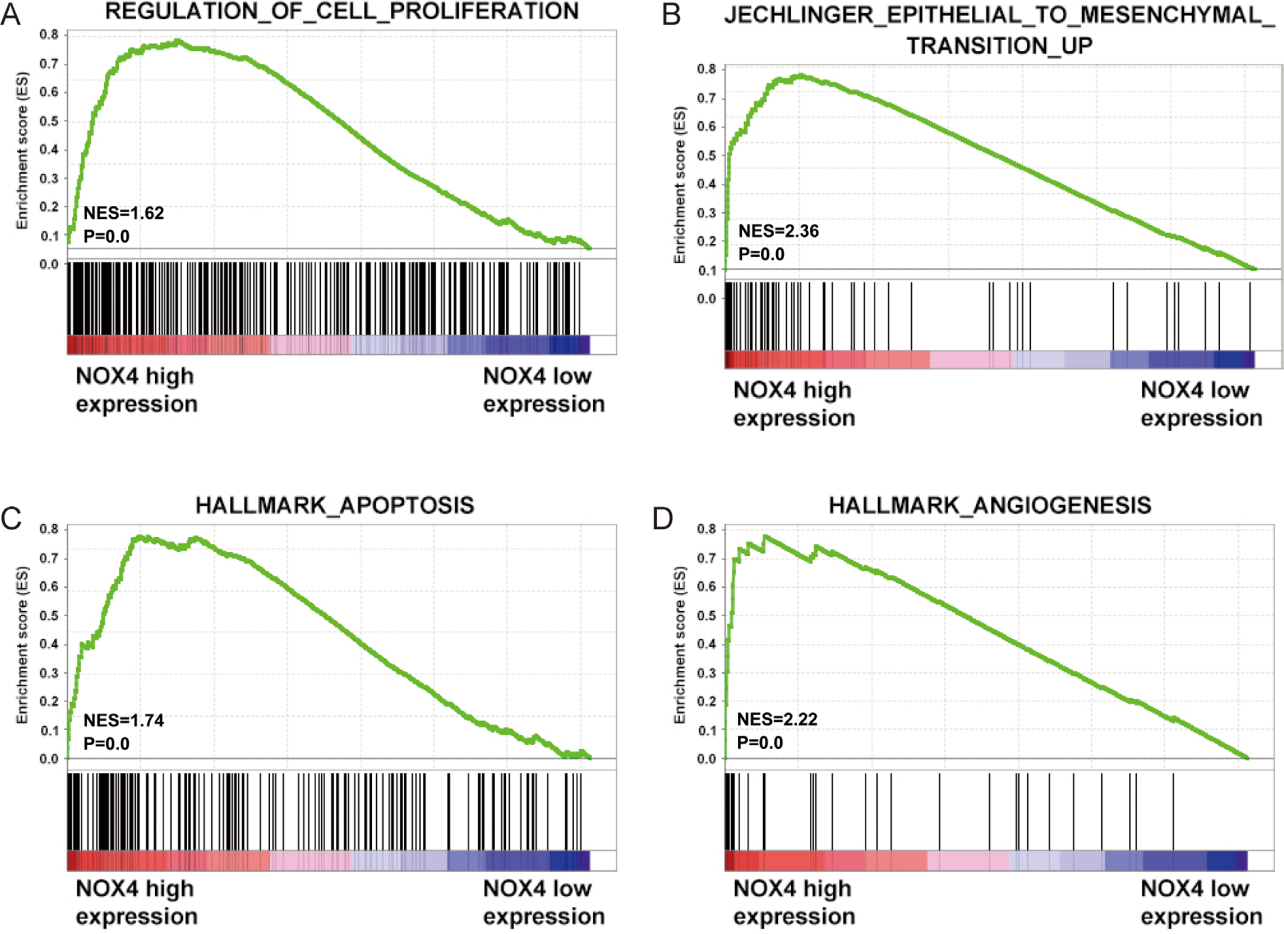
Biological functions of NOX4 in CRC (**A**–**D**) GSEA analysis on TCGA dataset showed the association of NOX4 expression with activity of the cell proliferation, metastasis, apoptosis and angiogenesis signaling pathway. The enrichment score (ES, green line) means the degree to which the gene set is overrepresented at the top or bottom of the ranked list of genes.

**Table 4:** Univariate and multivariate analysis of prognostic parameters for survival in patients with CRC

Prognostic parameter	Univariable analysis		Multivariable analysis	
	HR (95% CI)	*P* value	HR (95% CI)	*P* value
Expression of NOX4 (high vs. low)	3.342 (1.679–6.652)	0.001*	2.919 (1.382–6.163)	0.005*
Age (< 65 vs. ≥ 65)	1.186 (0.643–2.186)	0.585	–	–
Sex (male vs. female)	1.310 (0.702–2.443)	0.396	–	–
Tumor location (right hemicolon vs. left hemicolon)	0.862 (0.472–1.574)	0.629	–	–
Tumor size (< 5 cm vs. ≥ 5 cm)	2.218 (1.155–4.259)	0.017*	2.158 (1.101–4.231)	0.025*
T classification (T1 vs. T2 vs. T3 vs. T4)	1.723 (1.007–2.949)	0.047*	0.967 (0.562–1.663)	0.903
Lymph node metastasis (Absent vs. Present)	1.707 (0.931–3.130)	0.084	–	–
Distant metastasis (Absent vs. Present)	5.269 (2.307–12.033)	0.000*	3.209 (1.321–7.799)	0.010*
Tumor differentiation (Well/moderate vs. poor)	1.475 (0.769–2.831)	0.242	–	–

The bold number indicates the *P*-values with statistical significance. HR: Hazard ratio; CI: Confidence interval.

### Bioinformatics prediction of NOX4 function in CRC

To gain insight into the biological pathway involved in CRC pathogenesis stratified by the median of NOX4 expression level, GSEA analysis was performed in TCGA datasets. Enrichment plots of GSEA showed that the gene signatures of cell proliferation, apoptosis, metastasis and angiogenesis in patients with higher NOX4 expression were more active than those in patients with lower NOX4 expression (Figure 4). Thus, these analyses suggested that overexpression of NOX4 may be a key regulator in CRC.

### NOX4 knockdown inhibits proliferation and induces apoptosis of CRC cells

**Figure 5:**
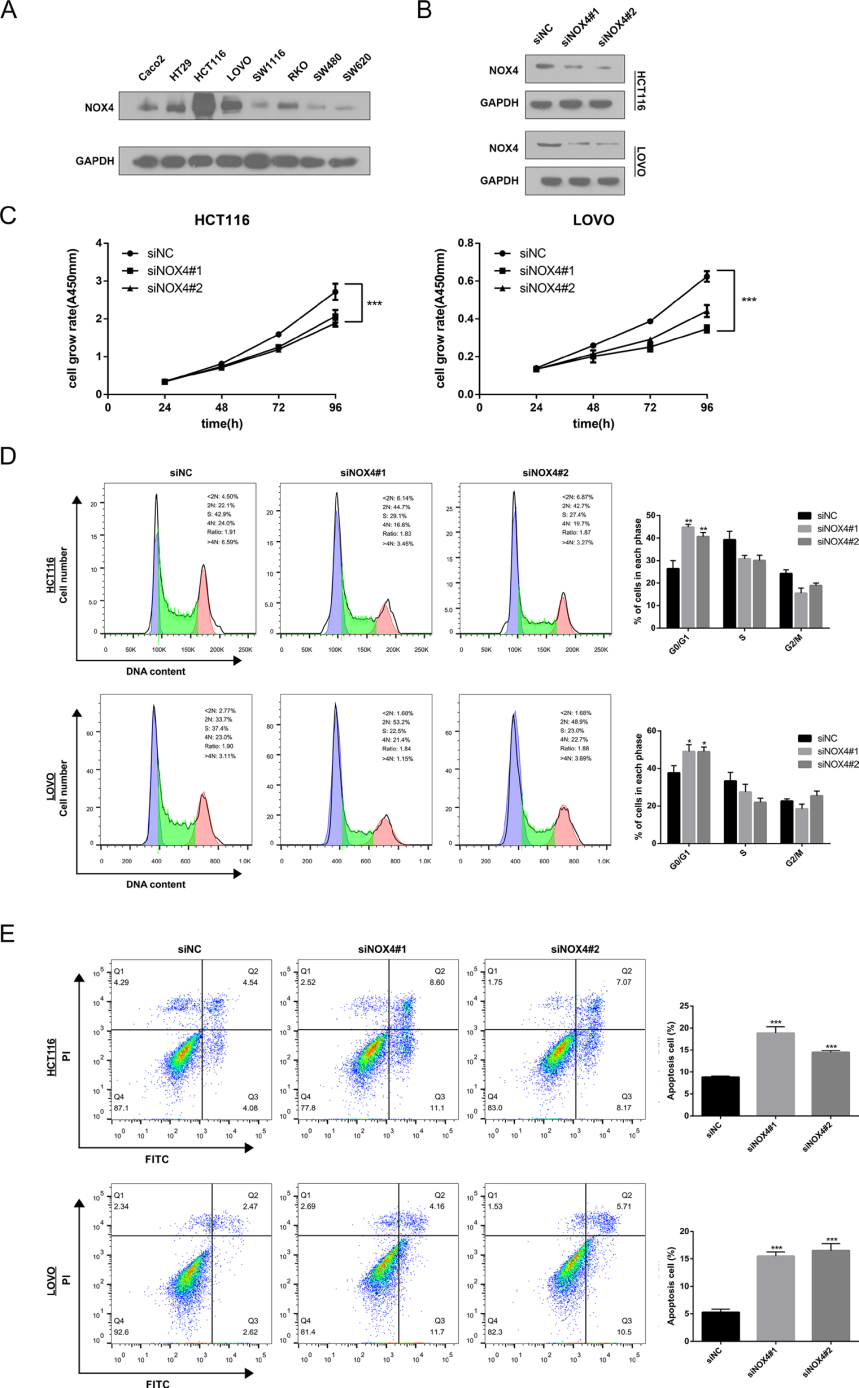
Knockdown of NOX4 inhibits the growth of CRC cell (**A**) NOX4 expression was measured in eight CRC cell lines by western blot. (**B**) Western blot analysis of NOX4 expression in NOX4-depleting HCT116 and LOVO cells. GADPH was used as the loading control. (**C**) The cell growth rates were determined by CCK-8 proliferation assays at various time points. (**D**) Cell-cycle arrest after knockdown of NOX4 in HCT116 and LOVO cells was assessed by flow cytometry. (**E**) Flow cytometry analysis of cell apoptosis in HCT116 and LOVO cells with or without NOX4 depletion. Results shown are the mean ± SD of triplicate determinations from three independent experiments (*P < 0.05, **P < 0.01, and ***P < 0.001).

To validate the GSEA analysis of NOX4, we assessed the tumorigenic ability of NOX4 in CRC cells by CCK-8 proliferation assays. First, we determined NOX4 expression in eight CRC cell lines at protein level. Our data showed expression of NOX4 varied in these cell lines, with the highest expression in HCT116 and LOVO cells (Figure 5A). Therefore, we transfected HCT116 and LOVO cells with NOX4-siRNAs, and western blot analysis verified NOX4 protein was markedly downregulated in both HCT116 and LOVO cells transfected with the two siRNAs (Figure 5B). Figure 5C shows that the proliferation of HCT116 and LOVO cells transfected with siRNA#1 or #2 was significantly inhibited compared with that of the control group, indicating that NOX4 contributed greatly to the proliferation of the CRC cells. In addition, we also examined the effects of NOX4 on CRC cell-cycle progression and apoptosis. As illustrated in Figure 5D and 5E, knockdown of NOX4 significantly induced G0/G1 phase arrest in HCT116 and LOVO cells, and the apoptotic indexes of NOX4 -silenced groups were significantly higher than those of control cells.

**Figure 6:**
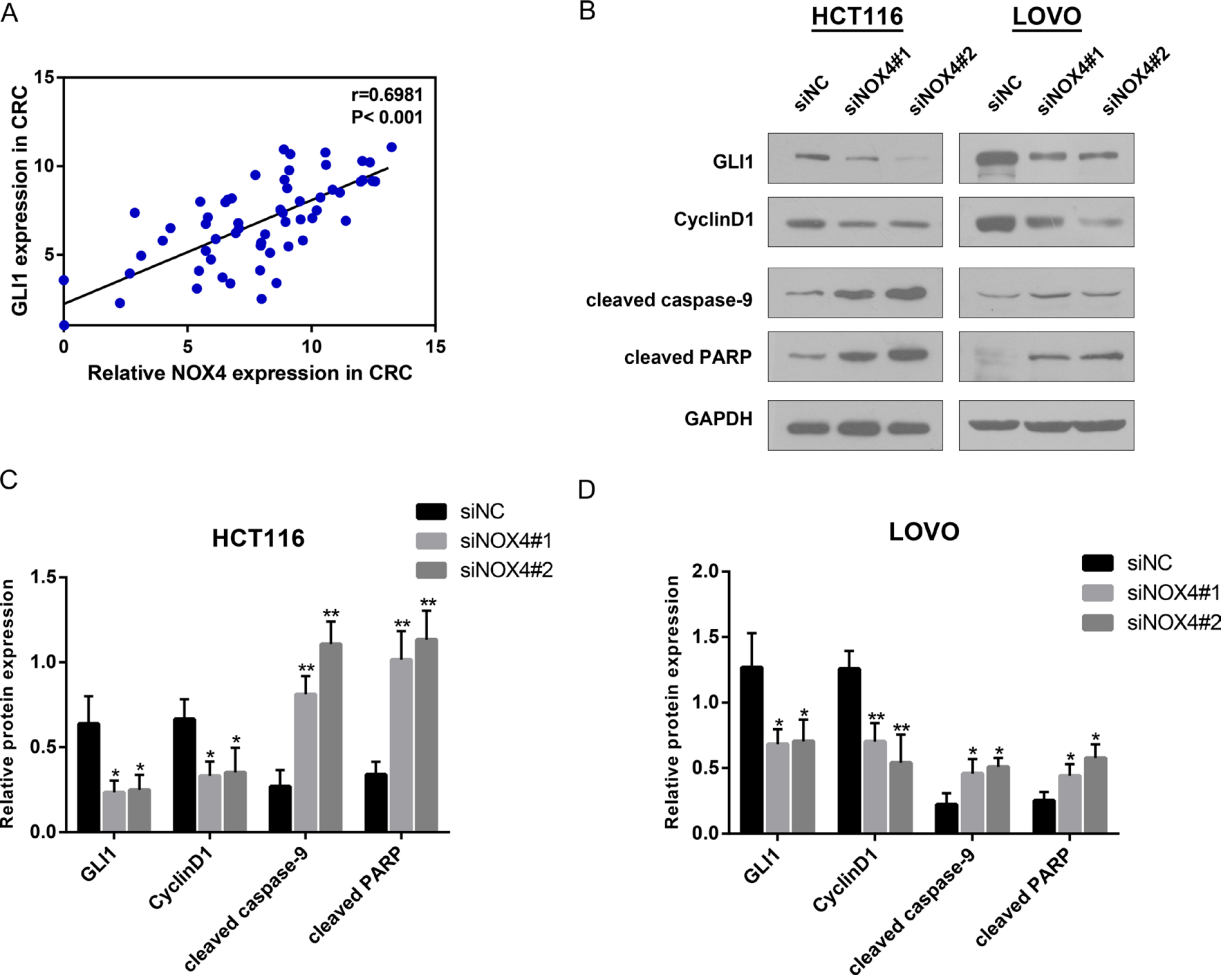
NOX4 positively associates with the growth related genes (**A**) Correlation analysis reveals a positive correlation between NOX4 and GLI1 mRNA expression in CRC tissues (*r* = 0.6981, *P* < 0.001). (**B**–**D**) Analysis of GLI1, Cyclin D1, cleaved caspase-9 and cleaved PARP expression by western blot in HCT116 and LOVO cells. GAPDH was used as internal control. Results shown are the mean ± SD of triplicate determinations from three independent experiments (*P < 0.05, **P < 0.01, and ***P < 0.001).

According to the proliferation and apoptosis pathways in GSEA, we found GLI1, a well-known transcription factor involved in the growth of many human tumours [21–23], was active in patients with higher NOX4 expression. Correlation analysis according to RT-PCR data indicated a notable positive relationship between GLI1 and NOX4 in CRC tissues (Figure 6A), and the protein level of GLI1 was down-regulated in both cell lines when NOX4 was silenced. In addition to GLI1 down-regulation, Cyclin D1, which is a critical regulator for cell cycle progression, and apoptosis-related proteins, cleaved caspase-9 and cleaved PARP, were all altered in NOX4-knockdown cells compared with their respective controls (Figure 6B and 6D). Collectively, these data suggest that as an oncogene NOX4 is required for the efficient cell growth in CRC cells.

### NOX4 influences CRC cell migration, invasion and epithelial-mesenchymal transition

**Figure 7:**
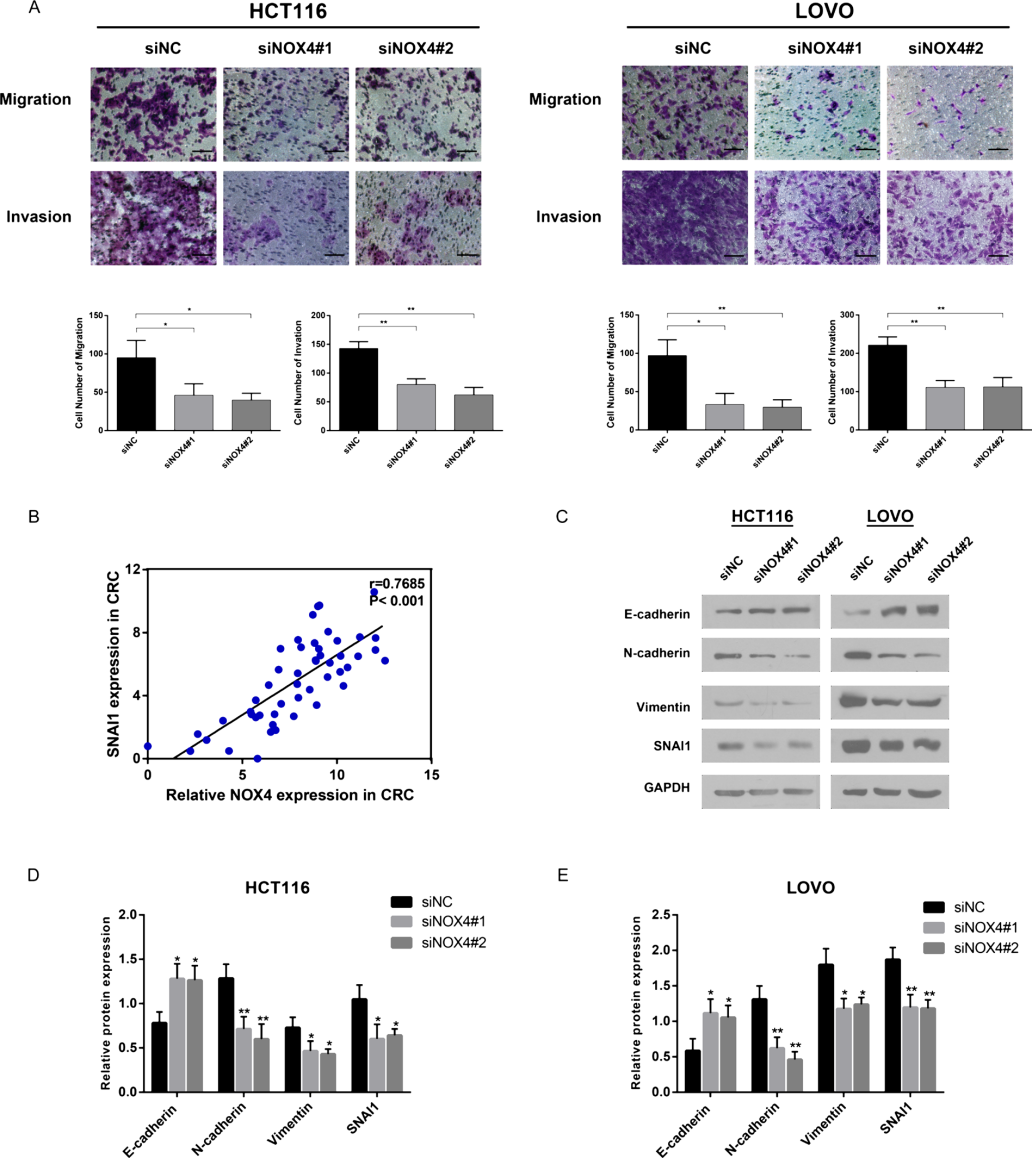
NOX4 promotes tumor invasion and metastasis *in vitro* by inducing EMT (**A**) Migration and invasion abilities were measured by transwell chamber assay in HCT116 and LOVO cells. Results were quantitated by counting invasive cells in five randomly selected high-power fields for three replicates. Scale bar = 100 μm. (**B**) RT-PCR revealed that a significant positive correlation between NOX4 and SNAI1 in CRC tissues (*r* = 0.7685, *P* < 0.001). (**C**–**E**) Protein level of epithelial markers (E-cadherin) and mesenchymal markers (N-cadherin, Vimentin, SNAI1) was measured by western blot in HCT116 and LOVO cells normalized to GAPDH expression. Results shown are the mean ± SD of triplicate determinations from three independent experiments (*P < 0.05, **P < 0.01, and ***P < 0.001).

We discovered NOX4 overexpression was significantly associated with CRC metastasis depending on clinicopathological data and GSEA analysis, thus the role of NOX4 in tumor cell migration and invasion was investigated. Transwell migration assay showed that the migration rate of control cells was greater than that of NOX4-depleted CRC cells by the two siRNAs. Likewise, transwell invasion assay indicated that NOX4-siRNA transfectants displayed a lower ability of invasion compared with negative control (Figure 7A). These results indicate that NOX4 deficiency impairs the migration and invasion ability of CRC cells. Based on GSEA analysis, the epithelial-mesenchymal transition (EMT)-related gene signatures were more active in patients with higher NOX4 expression than in those with lower NOX4 expression. RT-PCR revealed that a significant positive correlation between NOX4 and EMT-related transcription factor SNAI1 in CRC tissues (*r* = 0.7685, *P* < 0.001, Figure 7B). Furthermore, western blot data revealed that both HCT116 and LOVO cells transfected with the two siRNAs expressed high levels of E-cadherin, whereas mesenchymal biomarkers of N-cadherin, Vimentin and SNAI1 were decreased (Figure 7C and 7E). Altogether, these results strongly demonstrate NOX4 promotes the invasiveness of CRC through EMT processes.

## DISCUSSION

The human NOX4 gene is a 578 amino acid, six transmembrane domain flavocytochrome located on chromosome 11q14.2-q21. As a key oxygen sensor, the biological function of NOX4 is generating ROS by transporting electrons from cytosolic NADPH across biological membranes to molecular oxygen [24]. It was first identified as kidney-specific gene product by Shiose et al. in 2001 [25]. Accumulating evidence has revealed that NOX4 plays an important role in several types of tumor cells, leading to different outcomes. Depletion of NOX4 can induce apoptosis in pancreatic cancer cells through the AKT–ASK1 pathway [26]. NOX4-generated ROS are required for transformation phenotype of melanoma cells and contribute to melanoma growth [27]. However, the regulation and function of NOX4 in colorectal carcinoma and its relationship with clinicopathological features including prognosis of CRC patients remains to be elucidated.

In this study, we confirmed that NOX4 was markedly increased in CRC tissues compared with corresponding adjacent non-cancerous tissues by RT-PCR and Western blot. These findings are consistent with Oncomine analysis, which highlights the same trends in CRC. According to the meta-analysis across six Oncomine datasets, we also revealed NOX4 was one of the mostly up-regulated genes in colon cancer. Moreover, we extended our analysis to a non-overlapping cohort of 82 patients and found that NOX4 overexpression in tissue sections is strongly associated with T classification, lymphatic metastasis and distant metastasis. Importantly, patients with NOX4 overexpression had significantly shorter survival time than those with low expression, which was also confirmed by GSE14333, GSE17536 datasets from GEO. Cox proportional hazard regression analysis further identified NOX4 as an independent factor for poor prognosis. Taken together, these observations strongly indicated that NOX4 may be a cancer promoter for CRC and may play an important role in CRC progression.

The biological function of NOX4 in CRC is still uncertain. Our study gives a new insight of NOX4 gene functional role in CRC. According to the predicting results obtained by bioinformatics, we detected that cell proliferation, metastasis, apoptosis and angiogenesis–related genes were enriched in NOX4 overexpression group compared with NOX4 lower expression group. After an extensive literature review on previous related studies [28–32], NOX4 is proven to involve in other solid tumors by impacting target genes or signal pathways, which is in accordance with our bioinformatics analyses. We next performed a series of function assays showed that CRC cell growth was significantly inhibited by knockdown of NOX4. To explore the molecular mechanism of NOX4 induced inhibition of CRC cell growth, GLI1, involved in the cell proliferation and apoptosis pathways of GESA, then caught our attention. It is the most powerful effector of Hedgehog pathway, which associates with the process of DNA damage, cell cycle arrest, cell apoptosis and tumor metastasis in a variety of tumors, including colon cancer [33–36]. Along with its downstream targets (Cyclin D1, cleaved caspase-9 and cleaved PARP), the protein level of GLI1 both in HCT116 and LOVO cells was altered by NOX4 silencing. Meanwhile, further study investigated that there was significantly positive correlation between the NOX4 and GLI1 expression, therefore, it is reasonable to hypothesize NOX4 may target the Hedgehog pathway to induce CRC cell survival, but we need further experimental data to support this hypothesis.

Metastasis is a leading cause of cancer-related death, resulting in a poor prognosis. Previous research has shown that Schisandrin B, a novel inhibitor of NOX4, inhibits EMT and attenuates breast cancer cell metastasis [37]. In our study, we discovered the migration and invasion abilities of HCT116 and LOVO cells were attenuated after knockdown of NOX4, which was supported by our clinical data and GSEA analysis. Besides, further investigation revealed that suppressed expression of NOX4 induced EMT by elevating expression of the epithelial marker E-cadherin and reducing expression of the mesenchymal marker SNAI1, Vimentin and N-cadherin. To sum up, these data indicated that NOX4 may drive EMT in CRC cells, resulting in metastasis.

To date, there have been few studies on the role of NOX4 in CRC. Although this is the first report on the role of NOX4 in CRC, detailed mechanisms remain to be elucidated. We next plan to carry out further research on NOX4 molecular mechanism both *in vitro* and *in vivo*, especially the biological role of NOX4 in CRC angiogenesis, considering the limitation of this first study with respect to its oncogenic mechanisms in CRC. Besides, due to the limited sample size used in this study, future studies may be performed with larger sample size to conduct in-depth studies about the characteristics of NOX4 in CRC patients.

In conclusion, this is a preliminary study to investigate the role of NOX4 in CRC. We use computational analysis of high volume data to predict the oncogenic role of NOX4, patient survival and novel biological mechanisms. All our results highlight NOX4 can be considered a novel prognostic or therapeutic biomarker in patients with CRC. Therefore, we suggest that NOX4 could contribute to develop a novel effective strategy in CRC diagnosis and therapy.

## MATERIALS AND METHODS

### Clinical specimens

Human CRC tissue microarrays (TMA) including 82 pairs of primary CRC and corresponding non-tumor tissues were obtained from Renji Hospital, affiliated to the Shanghai Jiaotong University School of Medicine, between June 2009 and November 2009. None of the patients received preoperative chemotherapy, radiotherapy, or other tumor-specific therapies. A total of 82 cases were followed up by interview in the clinic or by telephone. The follow-up time ranged from 1 to 77 months, which was calculated from the date of surgery to CRC-related death, or the last follow-up time point (December 7, 2015). Additional 56 pairs of freshly-frozen samples were collected from CRC patients who underwent surgery at our hospital from February 2015 to August 2015, to test the mRNA and protein levels of NOX4 expression.

All samples in Oncomine, GEO and TCGA dataset have been collected and used following strict human subjects protection guidelines. The study was approved by the Ethics Committee of Ren Ji hospital, School of Medicine, Shanghai Jiao Tong University, and written informed consent was obtained from all participants involved in the study.

### Bioinformatics analysis

To determine the expression pattern of NOX4 in CRC, the datasets in Oncomine Cancer Microarray database (https://www.oncomine.org) were used. The datasets of corresponding clinical data used in this study were downloaded from the Gene Expression Omnibus (GEO) and TCGA website (https://tcga-data.nci.nih.gov/tcga/) following approval of these projects by the consortium. Data extraction was performed with R 3.0.2 software.

To gain further insight into the biological pathways involved in CRC pathogenesis through NOX4 pathway, a gene set enrichment analysis (GSEA) was performed. The canonical pathways gene sets (c2.cp.v4.0.symbols.gmt) from the Molecular Signatures Database-MsigDB (http://www.broad.mit.edu/gsea/msigdb/index.jsp) were used for enrichment analysis. Only gene sets represented by at least 15 genes were retained [38].

### Cell lines and transfection

All colorectal cancer cell lines were purchased from the American Type Culture Collection, and cultured in RPMI 1640 medium (Gibco, Gaithersburg, MD, USA) supplemented with 10% FBS, 100 U/ml penicillin and 100 μg/ml streptomycin in a 5% CO_2_ atmosphere at 37°C. Small interfering RNAs (siRNAs) targeting human NOX4 were transiently transfected into CRC cells using Genmute reagent (SignaGen, USA), whereas nontargeting control siRNA was used as negative controls. The siRNAs were purchased from Genepharm Technologies (Shanghai, China) and their sequences were designed as follows: #1: sense, 5′-GCCUCAGCAUCUGUUCUUATT-3′ and antisense, 5′-UAAGAACAGAUGCUGAGGCTT -3′; #2: 5′-CCAGGAGAUUGUUGGAUAATT-3′ and antisense, 5′-UUAUCCAACAAUCUCCUGGTT-3′.

### RNA extraction and quantitative real-time PCR

Total RNA was extracted by Trizol reagent (Takara, Japan), and 1 μg of total RNA was reverse transcribed using PrimeScript RT-PCR kit (Takara, Japan) according to the manufacturer's guidelines. RT-qPCR (Applied Biosystems, Grand Island, NY, USA) was performed to detect the expression of each specific gene with the SYBR-Green method (Takara, Japan). The 2−ΔΔCt method was used to quantify the relative NOX4 expression levels and normalized to 18S RNA. The primers used for RT–PCR in this study were as follows: NOX4, sense (5′- TGTGCCGAACACTCTTGGC-3′)and antisense (5′-ACATGCACGCCTGAGAAAATA -3′); GLI1, sense (5′- GAAGTCATACTCACGCCTCGAA -3′) and antisense (5′- CAGCCAGGGAGCTTACATACAT-3′); SNAI1, sense (5′- TCGGAAGCCTAACTACAGCGA-3′)and antisense (5′- AGATGAGCATTGGCAGCGAG-3′);18s RNA, sense (5′- CGGACAGGATTGACAGATTGATAGC-3′) and antisense (5′- TGCCAGAGTCTCGTTCGTTATCG-3′).

### Western blotting

Total protein from freshly-frozen tissues was extracted by RIPA (Beyotime, China) containing a protease inhibitor mixture (KangChen, China). Protein concentrations were quantified using a BCA Protein Assay Kit (Pierce Biotechnology). Protein samples were fractionated on 10% SDS-PAGE and transferred onto nitrocellulose membranes (Millipore). After blocking with 5% fat-free milk, the membranes were incubated with primary antibodies at 4°C overnight. The membranes were labelled with HRP-conjugated secondary antibodies for 1 h at room temperature and the immunoreactive signals were detected using Super Signal West Femto Maximum Sensitivity Substrate (Thermo Fisher, USA).

The primary antibodies were NOX4 (1:1000, ab133303, Abcam), GLI1 (1:1000, ab134906, Abcam), GAPDH (1:1000, WeiAo, China China). Other antibodies were all purchased from Cell Signaling Technology Inc (USA). Secondary antibodies were HRP-conjugated goat anti-rabbit antibody (1:5000, WeiAo, China).

### Immunohistochemical staining and evaluation

A traditional immunohistochemical (IHC) staining protocol was used in this study. Briefly, the tissue microarray section was deparaffinized in xylene and rehydrated with different concentrations of alcohol. Then the section was treated with 3% hydrogen peroxide, followed by antigen retrieval with 10 mM citrate buffer (pH 6.0) with microwave. After being blocked with 10% goat serum for 30 min, the section was incubated with anti-NOX4 antibody (rabbit polyclonal antibody, 1:100 dilution, ab133303, Abcam) overnight at 4°C, followed by a peroxidase-labeled secondary antibody.

NOX4 staining was semiquantitatively assessed using a grade scoring system according to the intensity of staining (scored as: 0, no staining; 1, weak staining; 2, moderate staining; 3, strong staining) and the percentage of positive tumor cells (scored as: 0, none; 1, 1%–29%; 2, 30%–69%; 3, > 70%). Total IHC score of each tissue sample was calculated to determine the cut-off value for low and high expression group by multiplying staining intensity score to the positive tumor cell score. And the final score, which ranged from 0–9, was defined as follows: 0, negative; 1–3, weak; 4–6, moderate; and > 6, strong. Therefore, NOX4 expression was thus sorted into 2 categories: high level (grades 4–9) and low level (grades 0–3). NOX4 staining was scored by two independent pathologists in a blinded manner.

### Cell proliferation assay, cell-cycle and apoptosis analysis

The viability of cells was assessed by Cell Counting Kit-8 (CCK8, Dojindo Molecular Technologies, Japan) according to the manufacturer's instructions. Tumor cells were seeded onto the 96-well plates at a density of 3 × 10^3^ cells per well. CCK8 solution was added to each

well at the time points of 24 h, 48 h, 72 h, and 96 h. The plate was examined at 450 nm absorption with a microplate reader after incubating for 2 hours at 37°C.

Cell cycles were examined using propidium iodide (PI) (BD Biosciences). Cells were washed with cold PBS and fixed in 75% ethanol −20°C overnight, and then incubated with PI for 15 minutes before flow cytometric analysis. For measuring the extent of apoptosis, CRC cells were analyzed through flow cytometry assay using an Annexin V FITC/PI double stain assay (BD Biosciences) following the manufacturer's protocol.

### Migration and invasion assays *in vitro*

Cell migration and invasion were carried out using 8-μm transwell filters (Millipore, USA). 1 × 10^5^ cells in serum-free medium were seeded into the upper chamber containing an uncoated or fresh Matrigel (diluted in 1:4 with serum-free medium) (BD Biosciences)-coated membrane. Meanwhile, 600 μL corresponding medium containing 20% FBS was applied to the lower chamber. At the end of the assay, cells that did not migrate or invade the filter were wiped with a cotton swab, cells on the bottom surface of the membrane were fixed and stained with 4 % formaldehyde and 0.1% crystal violet, respectively. The number of cells from five random microscope fields per filter was counted under an inverted microscope at 200× magnification.

### Statistical analysis

All the statistical analyses were carried out using the SPSS 17.0 software (SPSS Inc, Chicago, IL, USA) and GraphPad Prism 5 (San Diego, CA) software. The relationship between the NOX4 expression and clinicopathological factors was analyzed by chi-squared test. Survival curves for NOX4 expression were generated using the Kaplan-Meier method and compared using the log-rank test. Univariate and multivariate analyses were performed using Cox proportional hazards model to identify the prognostic factors. Pearson's correlation coefficient was performed to investigate the linear relationship of two variables. *P* values < 0.05 were considered statistically significant.

## Abbreviations

CRC: colorectal carcinoma
NOX4: NADPH oxidase 4
ROS: reactive oxygen species
TMA: tissue microarrays
IHC: immunohistochemical
GEO: Gene Expression Omnibus
GSEA: Gene Set Enrichment Analysis
TCGA: The Cancer Genome Atlas
EMT: epithelial-mesenchymal transition
